# CST3 alleviates bilirubin-induced neurocytes’ damage by promoting autophagy

**DOI:** 10.1515/tnsci-2022-0314

**Published:** 2023-10-16

**Authors:** Zhenkun Li, Yating Du

**Affiliations:** Department of Anesthesiology, Beijing Friendship Hospital, Capital Medical University, No. 95 Yong-An Road, Xi-Cheng District, Beijing, 100050, People’s Republic of China; Beijing Clinical Research Institute, Beijing Friendship Hospital, Capital Medical University, No. 95 Yong-An Road, Xi-Cheng District, Beijing, 100050, People’s Republic of China

**Keywords:** hyperbilirubinemia, unconjugated bilirubin, cystatin C, autophagy

## Abstract

High concentrations of unconjugated bilirubin (UCB) have toxic effects. The aim of our study was to find a way to elevate UCB tolerance or inhibit its toxicity in neurocytes. It has been reported that cystatin C (CST3) concentrations have a significant positive correlation with total bilirubin (TB) levels and a negative correlation with albumin levels. In addition, CST3 can directly bind UCB, decrease human umbilical vein endothelial cells’ permeability, improve blood–brain barrier integrity after ischemic brain injury in mice, and induce autophagy. We hypothesized that CST3 could increase the solubility of UCB, decrease permeability of neurocytes, induce autophagy of neurocytes, and alleviate bilirubin-induced damage. To verify our hypothesis, we measured TB and conjugated bilirubin levels, and the permeability and autophagy of neurocytes treated with UCB and CST3. Our findings suggest that CST3 can protect against UCB-induced damage in neurocytes and that autophagy played an important role in this process.

## 1 Introduction

Hyperbilirubinemia, presenting as jaundice, refers high serum bilirubin levels caused by various conditions [1]. Total bilirubin (TB) includes unconjugated bilirubin (UCB) and conjugated bilirubin (CB) [2]. UCB combines with albumin in the blood and is transported to the liver, where it is conjugated into glucuronic acid bilirubin by UDP-glucuronosyltransferase 1A1 (UGT1A1) and becomes CB [3]. CB can be excreted through feces or urine and has minor effect on the body. Therefore, current research on injuries caused by bilirubin has mainly focused on UCB.

Sustained higher UCB levels can damage various organs [4]. Studies have shown that UCB can induce neurotoxicity [5], which is a serious consequence of neonatal hyperbilirubinemia and a major cause of neonatal brain injury worldwide [6].

Cystatin C (CST3) is a low molecular weight (≈13.3 kDa) protein produced by nucleated cells [7], and is the most important extracellular inhibitor of cysteine proteinases [8]. It has been reported that CST3 has protective effects in neurodegenerative diseases [9,10], but the role of CST3 on UCB-induced neurotoxicity has not been elaborated. Studies have shown that UCB could bind with cystatin [11,12]. Besides, human umbilical vein endothelial cells’ permeability decreases significantly after treatment with CST3 [13] and can improve blood–brain barrier integrity after ischemic brain injury in mice [14]. In addition, it has been reported that CST3 induced autophagy through the AMPK-mTOR pathway and plays a neuroprotective role [15]. In addition, CST3 knockout led to disordered autophagy in macrophages and ApoE-knockout mice [16]. We hypothesized that CST3 could reduce UCB toxicity via binding with UCB, decreasing the permeability of cells, or promoting autophagy.

## 2 Materials and methods

### 2.1 Hyperbilirubinemic mice

A total of 15 male C57BL/6 mice (6–8 weeks) were used in this study. Ten mice injected intravenously with 150 μg/g of UCB (total volume 100 μL). They then were evenly divided into two groups and injected intravenously with 5 μg/g of CST3 (total volume 100 μL) or PBS (total volume 100 μL). The animals were maintained under controlled conditions with a temperature of 24 ± 2°C, humidity of 55–65%, a 12 h light/dark cycle, and *ad libitum* access to standard laboratory diet and water. They were euthanized after 3 days and the tissues were fixed in 4% formalin or 2.5% glutaraldehyde.

### 2.2 Morris water maze

The mice need to find and stand on a platform submerged under 5 mm of water in a circular pool by observing four visual cues in different locations around the pool. On the training days, the mice were given a maximum of 60 s to find the platform, and recording was stopped when the mice remained on the platform for 5 s. Mice that did not find the platform within 60 s were guided to the platform and allowed to remain on it for 10 s. The training phase lasted for 4 days, during which the mice were placed in the pool four times every day. On the fifth day, the test was performed by placing the mice in the pool from which the platform had been removed and allowing them to explore for 60 s. The time and distance they swam in the platform quadrant or in the other three quadrants were measured.

### 2.3 Masson’s staining

For Masson’s staining, the brain samples were treated with hematoxylin for 5 min, with deionized water for 30 s three times, and with Masson’s dye (HT15, Sigma, USA) for 7 min. Then, the brain sections were treated with 2% glacial acetic acid solution and 10% molybdic acid for 5 min. After aniline blue staining for 5 min and gradient alcohol washing, neutral gum was used to seal the slide, and the section was observed under a microscope.

### 2.4 Bilirubin measurement

TB and CB were measured using TB assay kit (C019-1-1, Njjcbio, China) and a direct bilirubin assay kit (C019-2-1, Njjcbio, China), respectively. UCB content was defined by TB minus CB levels (µmol/L).

### 2.5 Cell culture

HT22 cells were cultured in RPMI-1640 medium (Sigma, USA). The medium was changed every 2–3 days. CST3 proteins were obtained from ProSpec and blocking peptides for CST3 were purchased from Abgent. The proteins were dissolved in double distilled water. UCB of 100 µg/mL was added to the cells to establish high UCB cell model.

### 2.6 Quantitative polymerase chain reaction (qPCR)

Total RNA was extracted from HT22 cells using TRIzol reagent (Tiangen, China). We synthesized cDNA using the FastQuant RT Kit (Tiangen, China) following the manufacturer’s instructions. qPCR was performed on an IQ5 thermal cycler (Bio-Rad, USA) using the following cycling conditions: predenaturation at 95°C for 15 min, 40 cycles of denaturation at 95°C for 10 s, annealing at 60°C for 15 s, extension at 72°C for 20 s, and 71 cycles of melt curve analysis at 60°C for 10 s. The following primers were used in reactions:

CST3 primer sequences: F CAACAAAGCCAGCAACGACA, R TCTTGGTACACGTGGTTCGG and β-actin primer sequences: F AGAGGGAAATCGTGCGTGAC, R CAATAGTGATGACCTGGCCGT.

### 2.7 Western blots

Proteins were extracted from the brain samples using a Proteins Extraction Kit (CWBIO, China) and quantified with BCA-Reagents (CWBIO, China). Proteins were separated by SDS–PAGE at 160 V on a 12% gel (CWBIO, China) for 1 h and then transferred to a 0.22 μm nitrocellulose filter membrane at 200 mA for 3 h. The membranes were incubated with autophagy related primary antibodies diluted as follows: anti-CST3 antibody (ab24327; Abcam, UK), anti-mTOR antibody (ab32028; Abcam, UK), anti-mTOR (phospho S2448) antibody (ab109268; Abcam, UK), anti-LC3 antibody (ab192890; Abcam, UK), anti-Beclin-1 antibody (3495, CST, USA) diluted 1:1,000, and anti-GAPDH antibody (ab181602; Abcam, UK) diluted 1:2,000. Membranes were then incubated with secondary antibodies diluted at 1:5,000. The membranes were washed and bands were visualized with enhanced chemiluminescence immunoblotting detection reagents (Thermo Fisher Scientific, USA). For autophagic protein, bafilomycin A1 was used at a final concentration of 200 nM and added during the last 3 h. Images were gotten by Image Lab Software (BIO-RAD, USA), and semiquantitative analysis was performed by normalizing the protein bands to the internal control GAPDH.

### 2.8 Cytotoxicity assay

We performed MTT (Solarbio, China) assays to examine the viability of HT22 cells treated with CST3 (100 pg/μL), UCB (170 µM), or bafilomycin A1 (200 nM and added during the last 3 h of treatment). Cells treated with solvent were used as the negative control. HT22 cells were plated at a density of 2,000 cells/well and allowed to adhere for 6 h, followed by treatment with CST3 protein and UCB. Wells containing 100 μL medium alone (without cells) were used as blank controls. MTT assays were performed after treatment for 48 h. The blank control served as baseline. Each experiment was repeated three times, and the results are presented as a percentage of viable cells calculated by the following equation: (mean absorbance of experimental well/mean absorbance of control well) × 100 = percentage of viable cells.

### 2.9 Enzyme-linked immunosorbent assay (ELISA)

The activity of P450 was determined using a P450 activity ELISA kit (Cyagen, USA) in accordance with the manufacturer’s instructions.

### 2.10 Cell permeability

HT22 cells (2 × 10^5^) were seeded onto polycarbonate cell culture inserts of a 2-well Transwell system (Costar) until they formed a complete monolayer. Then FITC–BSA was added to the upper chamber and the fluorescence was evaluated in the lower chamber after adding FITC–BSA for 24 h (excitation wavelength, 488 nm; emission wavelength, 525 nm).

### 2.11 UCB permeability

HT22 cells (2 × 10^5^) were seeded onto polycarbonate cell culture inserts of a 24-well Transwell system (Costar) until they formed a complete monolayer. Then 100 µg/mL UCB was added to the upper chamber and concentration of UCB in lower chamber was evaluated after 6 h.

### 2.12 Statistical analyses

Statistical analyses were performed using SPSS 22.0 (SPSS Inc., USA). After the normality test and variance homogeneity test of measured data, comparisons between different groups were performed using Student’s *t*-test or one-way ANOVA. Data are shown as mean ± SD. A *p*-value ≤0.05 indicated statistical significance.


**Ethical approval:** The research related to animals’ use has been complied with all the relevant national regulations and institutional policies for the care and use of animals. All animal experiments were approved by the Animal Experiments and Experimental Animal Welfare Committee of Beijing friendship hospital, Capital Medical University (21-2036).

## 3 Results

### 3.1 CST3 protects against UCB-induced injury in neurocytes

**Figure 1:**
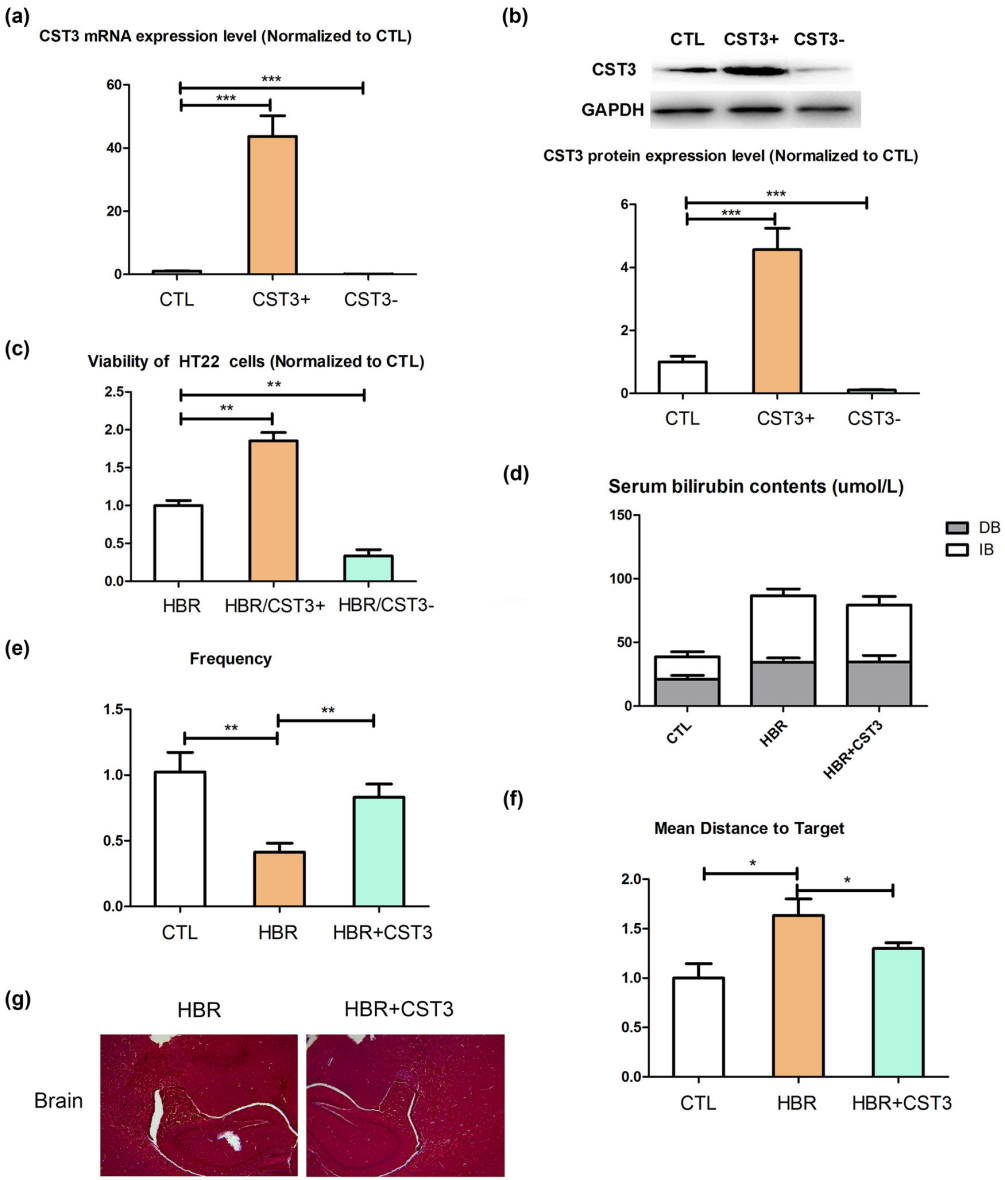
CST3 alleviates injury to neurocytes in the high-dose UCB group: (a) CST3 mRNA expression levels, (b) CST3 protein expression levels, (c) viability of HT22 cells treated with UCB and CST3, (d) serum bilirubin contents of hyperbilirubinemic mice, (e) frequency swimming across the platform, (f) mean distance to platform, and (g) Masson staining in the brains of hyperbilirubinemic mice treated with CST3. CTL: control group; HBR: high-dose UCB group; DB: direct bilirubin; IB: indirect bilirubin. *p ≤ 0.05; **p ≤ 0.01; ***p ≤ 0.001.

To investigate the role of CST3 on UCB toxicity, we established CST3 overexpression and knockdown HT22 cells (Figure 1a and b), which were treated with UCB. We found that the viability of HT22 cells overexpressing CT3 increased, while the viability of CST3 knockdown HT22 cells decreased (Figure 1c). We then established a hyperbilirubinemic mouse model (Figure 1d) and found the decrease of frequency swimming across the platform and increase the mean distance to platform in hyperbilirubinemic mouse, while CST3 could reverse this phenomenon (Figure 1e and f). Besides, it showed anesis of hippocampus neurocytic injury induced by UCB in the CST3-treated group compared to that in the PBS-treated group (Figure 1g).

### 3.2 Effects of CST3 on the solubility of UCB solubleness in a cell-free system and permeability in HT22 cells

**Figure 2:**
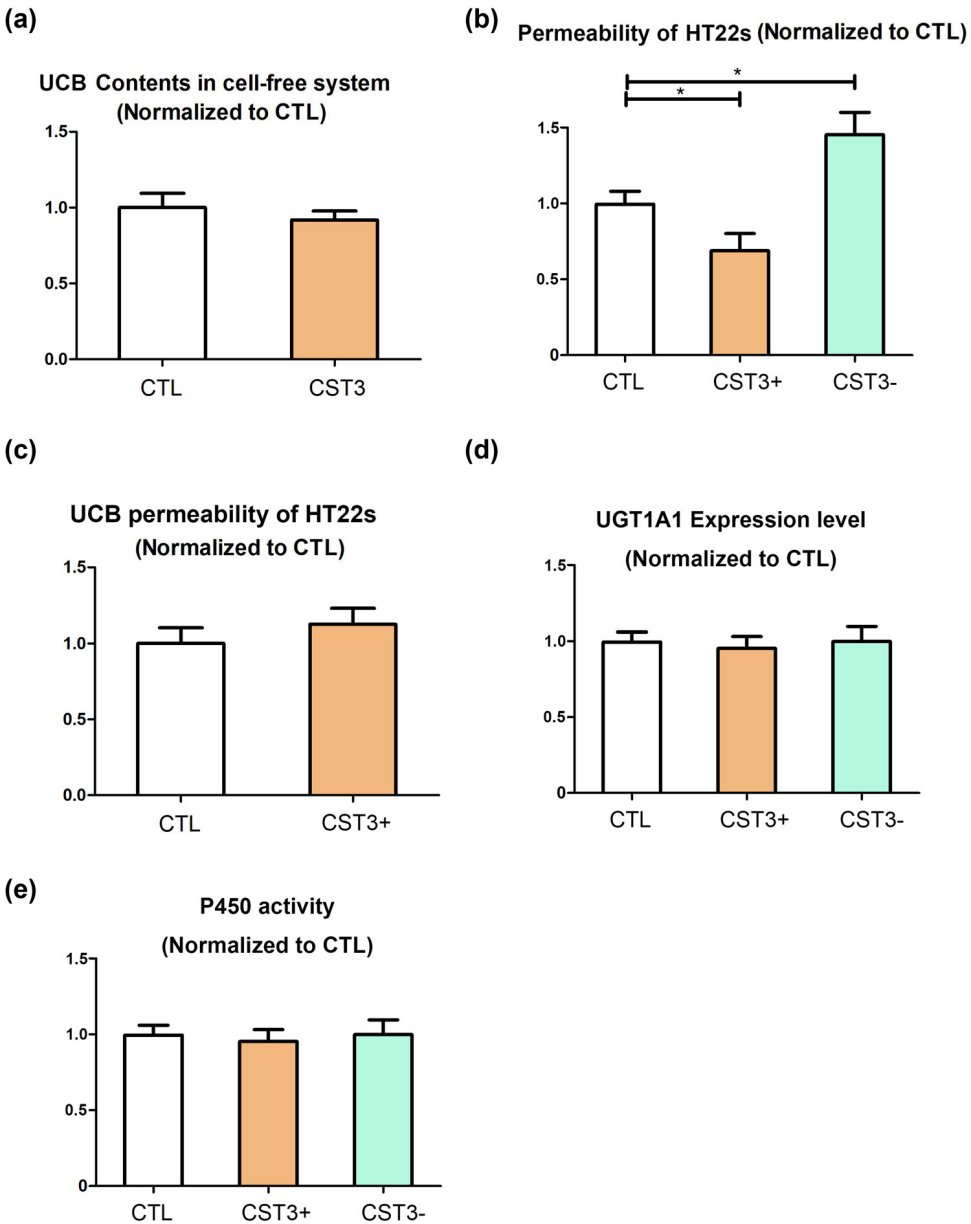
Effects of CST3 on UCB solubility in cell-free systems and permeability in HT22 cells: (a) UCB content in CTL and CST3 groups, (b) macromolecule permeability of HT22 cells of HBR and HBR + CST3 group, (c) UCB permeability in HT22 cells of HBR and HBR + CST3 groups, (d) expression level of UGT1A1, and (e) activity of cytochrome P450. CST3: adding CST3 protein; CST3+: CST3 overexpression; CST3−: CST3 knockdown. *p ≤ 0.05.

To investigate the mechanism of the protective effect of CST3 on hyperbilirubinemia, we measured the solubility of UCB, and found that CST3 could increase the solubility of UCB in a cell-free system (Figure 2a), but it did not have significant difference compared with CTL (CST3 solvent, PBS). In addition, we found that CST3 decreases the permeability of HT22 cells (Figure 2b), but UCB permeability of HT22 cells did not change (Figure 2c). In addition, we found that UCB did not change the expression level of UGT1A1 (Figure 2d) and the activity of cytochrome P450 (Figure 2e).

### 3.3 CST3 protects cells from UCB-induced damage by promoting autophagy

**Figure 3:**
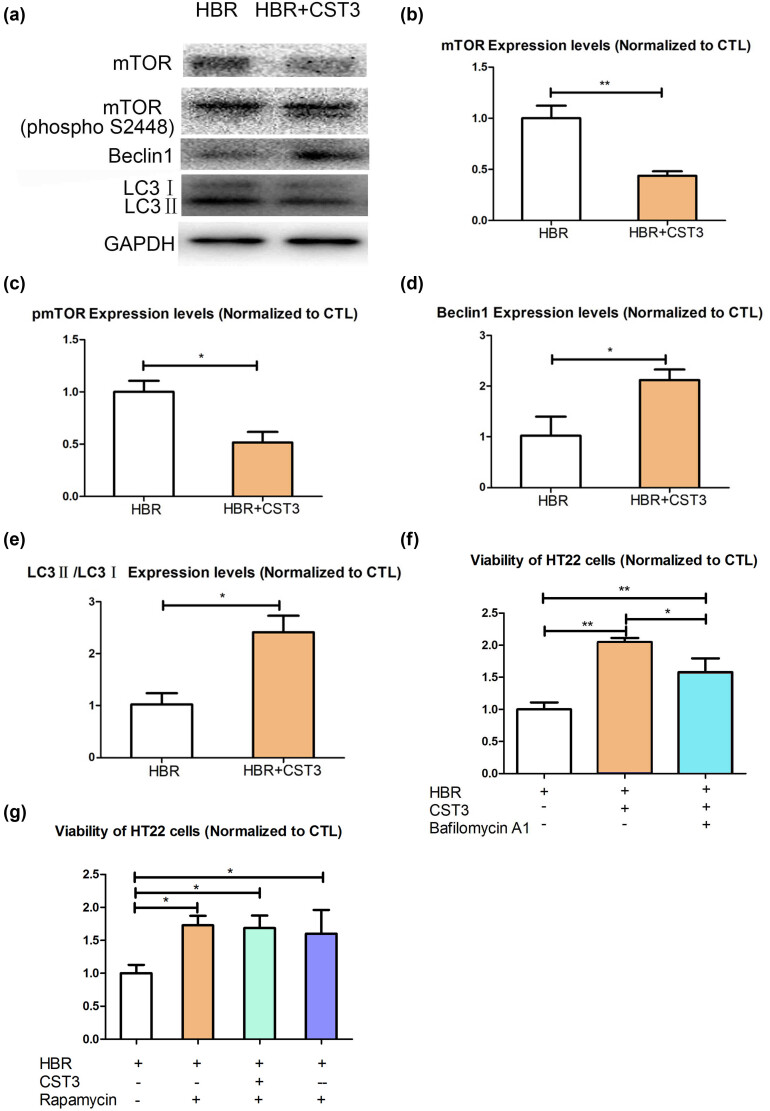
CST3 protects HT22 cells from UCB damage via promoting autophagy: (a) CST3 promotes HT22 cells autophagy, (b) mTOR, (c) pmTOR, (d) Beclin1, (e) LC3II/LC3I, (f) viability of HT22 cells treated with CST3 and autophagy inhibitor Bafilomycin A1, and (g) viability of HT22 cells treated with CST3 and autophagy activator rapamycin. HBR: high-dose UCB group; +: adding or overexpression; -: not adding; --: knockdown. *p ≤ 0.05; **p ≤ 0.01; ***p ≤ 0.001.

To further explore the mechanism of the protective effect of CST3 on hyperbilirubinemia, we studied the autophagy pathway and found that CST3 could enhance autophagy flux in HT22 cells (Figure 3a–e) and the protective effect could be inhibited by autophagy inhibitor bafilomycin A1 (Figure 3f). In addition, the effects of CST3 overexpression and knockdown vanished in the presence of autophagy activator rapamycin (Figure 3g).

### 3.4 CST3 alleviates UCB-induced injury in neurocytes by promoting autophagy

**Figure 4:**
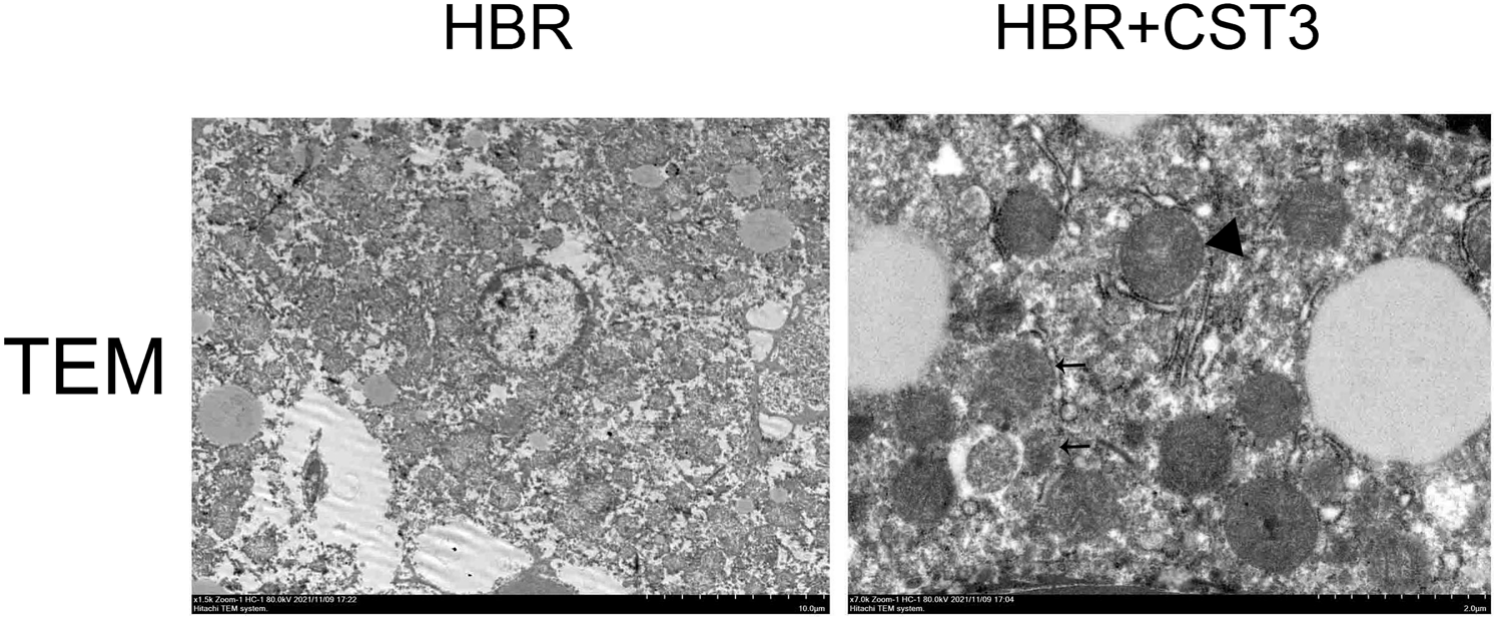
CST3 alleviates UCB-induced damage to neurocytes by promoting autophagy. Arrows: autophagosomes; triangle: mitochondria.

We found enhanced autophagy in the neurocytes of CST3-treated high concentration bilirubin (HBR) mice by transmission electron microscopy (Figure 4).

## 4 Discussion

We found that CST3 could reduce UCB toxicity by increasing the solubility of UCB, decreasing the permeability of HT22 cells, and promoting autophagy of HT22 cells. However, the effects of increasing solubility and decreasing permeability are intriguing. It might be because noncovalent binding between UCB and CST3 [11,12], which lead to the instability of the binding, and the permeability of UCB was almost unaffected by CST3. In addition, we found that UCB did not change the expression level of UGT1A1 and the activity of cytochrome P450. Autophagy played the most important role in CST3 inhibiting UCB neurotoxicity.

Effects of exogenous CST3 to enhance viability and autophagy of neurocytes suggest that CST3 might serve as a potential drug for the treatment of hyperbilirubinemia. In addition, CST3 is an endogenous protein and should have few side effects. Excessive CST3 is also excreted through urine.

In conclusion, we demonstrated that CST3 could alleviate UCB-induced damage to neurocytes by increasing UCB solubility, decreasing the permeability of neurocytes, and promoting autophagy, and that autophagy played the most important role in this process.
